# Materials Selection and Mechanism of Non-linear Conduction in Chalcogenide Selector Devices

**DOI:** 10.1038/s41598-018-37717-x

**Published:** 2019-02-12

**Authors:** Huanglong Li, John Robertson

**Affiliations:** 1 Department of Precision Instrument, Center for Brain Inspired Computing Research, Tsinghua University, Beijing, 100084 China; 2 Engineering Department, University of Cambridge, Cambridge, CB2 1PZ UK

## Abstract

The electronic structure and conduction mechanism of chalcogenide-based Ovonic threshold switches (OTS) used as selectors in cross-point memory arrays is derived from density functional calculations and quasi-Fermi level models. The switching mechanism in OTS is primarily electronic. This uses a specific electronic structure, with a wide tail of localized states below the conduction band edge. In amorphous GeSe_2−x_ the conduction band consists of Ge-Se σ*states with a low effective mass, and with a broad tail of localized Ge-Ge σ* states below this band edge. This leads to the OTS behavior. At high fields the electron quasi-E_F_ moves up through these tail states, lowering the conductivity activation energy, and giving the non-linear switching process. The 4:2 coordinated GeSe_2−x_ based alloys are the most favorable OTS material because they have the correct network connectivity to give a high electron mobility and lack of crystallization, a favorable band structure to produce the non-linear conduction, an optimum band gap, and with nitrogen or carbon alloying, a sufficiently low off-current.

## Introduction

Recently, the first commercial scale (128 Gb) non-volatile storage-class memory, the Xpoint, was introduced^1^. This is non-volatile, and claimed to be roughly x1000 faster than Flash memory, potentially cheaper, scalable and as fast as DRAM, and with a longer endurance than Flash. Using elemental analysis, the Xpoint was found to possess a GeSbTe-like phase change memory material^1^, and an upper layer of selector devices with a composition suggesting an Ovonic threshold switch (OTS)^1,2^. Generally, two-terminal memory devices, whether phase change random access memories (PCRAM) or resistive random access memories (RRAM), use a cross-point architecture as in the Xpoint, and need an additional non-linear conducting selector device in series to eliminate the conducting ‘sneak paths’ that occur between non-selected cells^3^.

Presently, selectors tend to use OTS devices consisting of Ge or Si chalcogenide-based semiconductors^4,5^. However, these materials have been much less studied than the analogous phase change materials themselves, despite their necessity in the multi-billion-$ PCRAM device business. The non-linear conduction of OTS devices occurs because they undergo an electronic transition between a low and high conducting state through a region of negative differential resistance^6^. They need a threshold current to stay in their high conducting state. They differ from the memory switches like PCRAM, which must be actively switched from one state to the other.

The materials requirements for OTSs and phase change materials (PCMs) differ. PCMs are semiconductors which undergo a rapid *structural* transition between their crystalline and amorphous phases, which have different underlying bonding in each phase^7,8^. For a rapid phase transition, PCMs should be poor glass formers. The best performing PCMs are typically tellurides such as Ge_2_Sb_2_Te_5_, based on the IV-VI semiconductors, with an average of 5 valence electrons per atom and an equi-molar GeTe stoiochiometry. PCMs are often resonantly bonded in their crystalline phases and have covalent molecular bonding in their amorphous (a-) phase^9–11^.

The OTS materials differ. Govoreanu^4^ recently compared the performance of different selector materials (not just chalcogenides) in terms of their conductivity nonlinearity versus maximum current density. The Ge-rich GeSe_2−x_ based OTS materials showed the highest drive current density and largest half-bias nonlinearity. Velea^5^ found that the favored chalcogenide selector materials centered on a-GeSe_x_, SiTe_x_ and SiSe_x_. The other key properties of selector materials are a fast transition between their low and high conductivity states, an absence of crystallization during operation, and a low off-current^4^. We now try to rationalize these observations in terms of material selection and phase stability diagrams. Here, we show that GeSe_x_, SiTe_x_ and SiSe_x_ each have similar band structures, which differ from the PCM GeSbTe, but that GeSe has the band structure leading to the best overall performance.

## Calculations

The early OTS materials were STAG glasses (Si,Te,As,Ge)^6,12–15^. These are p-type with a low hole mobility of about 2 × 10^−5^ cm^2^/V.s^6,13^, Table 1. More recent OTS materials such as the a-GeSe based semiconductors are n-type with much higher mobility of order 0.7 cm^2^/V.s (and hole mobilities of ~10^−2^ cm^2^/V.s)^16,17^. Given the desire for fast switching speeds, this mobility makes GeSe-type materials superior.

**Table 1:** Electron mobilities of various amorphous chalcogenides.

	Electron mobility (cm^2^/V.s)	reference
a-Se	10^–4^	^16, 18^
a-As_2_Se_3_	10^−5^	^19^
STAG glass	2 × 10^−5^	^6, 15^
a-Ge_0.03_Se_0.97_	10^−5^	^16^
a-GeSe_x_	2 × 10^−2^	^16^

a-Se consists of molecular units separated by van der Waals bonds, and this gives them a low mobility^16,18^. The difference in mobilities of Ge- and As-based materials arises from their different local structure, as shown in Fig. 1(a). The lower 3-fold coordination of As means that a-As_2_Se_3_ and As_2_Te_3_ consist of molecular units, separated by van der Waals bonds, giving low mobilities^19^ because they limited by inter-molecular hopping. This gives their band edge states a high effective mass^20^. On the other hand, the higher 4-fold coordination of Ge or Si in their 4:2 coordinated (silica-like) phases means that the Ge or Si alloys are fully connected networks^21^, with a lower effective mass and higher mobilities^16^.

**Figure 1:**
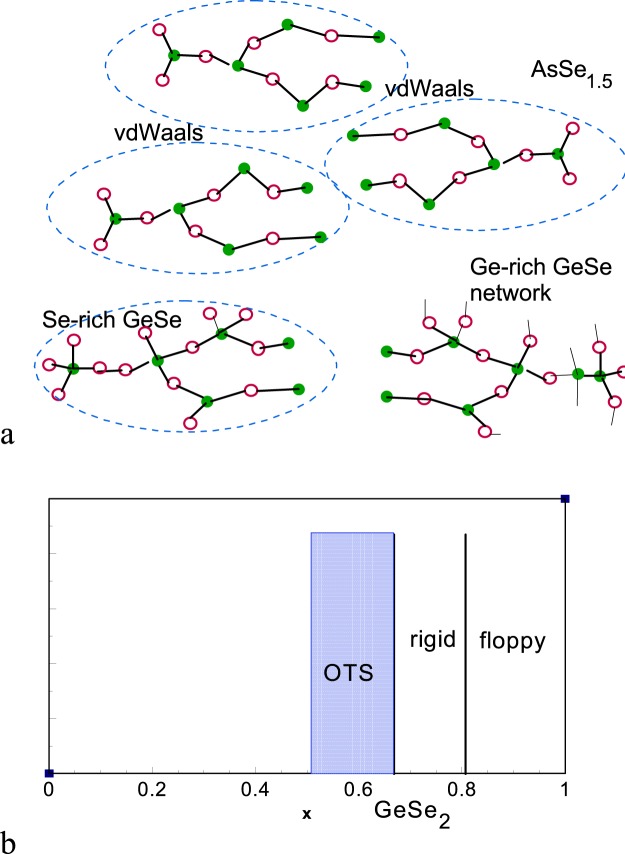
(**a**) Comparison of network structure of a-AsSe alloys, Se-rich and Ge-rich 4:2 coordinated a-GeSe alloys. (**b**) The amorphous Ge_1−x_Se_x_ alloys. The very Se-rich alloys were of interest for the floppy/rigid stiffness transition at x = 0.80^22^. Alloys of interest for OTSs devices lie at x = 0.55–0.66, Ge-rich of the GeSe_2_ composition. GeTe alloys of interest for PCM lie at x = 0.50^9^.

There are three compositional regimes for the Ge or Si chalcogenides, Fig. 1(b). The PCMs use equimolar compositions such as GeTe, which are consistent with their 3:3 coordinated rhombohedral or orthorhombic structures. At the other extreme are the very chalcogen-rich GeSe_2+x_ or GeS_2+x_ alloys consist of molecular units, like the a-As_2_Se_3_ case. These Se-rich GeSe_x_ alloys were once intensively studied because of interest in the rigidity transition^22^. The OTS materials themselves are 4:2 coordinated but with a higher Ge or Si content so that they form continuous, more highly connected molecular networks.

We now consider the design requirements of OTS devices. It is generally agreed since the work of Adler^6^ that the OTS transition is fundamentally of electronic origin. The strongly non-linear change in conductivity corresponds to a negative differential resistance. Ielmini^23,24^ modeled this as arising from Poole-Frenkel excitation from midgap trap states to the band edge states, causing a progressive filling of higher energy traps at high applied fields. However, Ielmini’s model applies more for high field conduction in wide-gap, weakly screened dielectrics like SiO_2_, rather than for hopping in localized states in moderately screened, disordered semiconductors like GeSe_2_. In this respect, Clima^25^ found that the Bohr radius of tail states in a-GeSe_x_ is 1–2 nm, much larger than those of defect states in SiO_2_.

**Figure 2:**
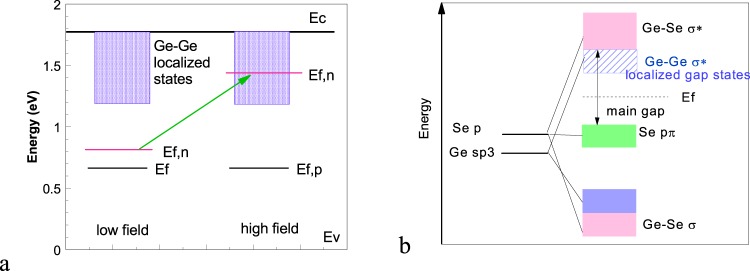
(**a**) Conduction model for the low field and high field condition for selector devices. E_f,n_ is quasi Fermi level for electrons which shifts for high fields. (**b**) Schematic band diagram of Ge_1+x_Se_2_ which gives rise to the conduction model of (**a**).

We therefore consider a different interpretation of Ielmini’s model, with localized tail states below a mobility edge as in Fig. 2(a)^26,27^. We use the concept of quasi Fermi levels from photoconductivity. At low fields, the electron and hole quasi-Fermi levels lie at the same energy, near midgap. The conductivity is very small, because the activation energy ∆E from E_F_ to the mobility edge is large, half the mobility gap. As the field increases, the occupation of tail states causes the electron and hole quasi Fermi levels to diverge. In particular, the electron E_F,n_ rises up through the broad band of conduction band (CB) tail states, while the hole E_F,p_ remains near midgap because the valence band (VB) tail is quite sharp. The n-type activation energy will decrease sharply, and the conductivity will increase rapidly. This leads to a strongly non-linear dependence of conductivity on applied field. The concept of quasi-Fermi level implies that E_F_ is in equilibrium, so the rate of excitation to these higher states due to ‘upwards’ hopping equals the rate of thermalization back to lower states.

We now design a chalcogenide band structure to satisfy these requirements. The overall band structure in Fig. 2(b) consists of three types of bands; the bonding (σ) states well below the VB top, the chalcogen lone pair pπ states forming the upper VB, and the antibonding (σ*) states forming the CB^28–30^. The pπ band is non-bonding, of high effective mass m*, and it does not have a wide tail state distribution. The GeSe_2_ CB consists of σ* states with much lower effective mass. These are Ge sp^3^/Se p states. The band edges of such Ge-Se σ* states are sensitive to disorder and can be localized because they are sp^3^-like as in a-Si, as noted by Hosono^31^. Overall, the band gap has tail states primarily on the CB side, and few on the VB side. This is the configuration required by Fig. 2(a). This same overall band layout holds for a-GeSe, a-SiTe and a-SiSe.

We now consider the effects of off-stoichiometry from the stoichiometric GeSe_2_ composition. The OTS design should avoid Se-rich compositions, as they create Se-site defects which can mediate slow, structural transitions and which go against the concept of electronic-only transitions. Also, they can introduce Se-site disorder and band tails on the VB side, which go against the desire for unipolar conduction.

An excess of Ge from GeSe_2_ will introduce Ge-Ge bonds. The σ* state of an isolated Ge-Ge bond will generally lie in the CB. Nevertheless, higher Ge excess will lead to a band tail of localized Ge-Ge σ* states appearing beneath the main CB of Ge-Se σ* states. The width of this Ge-Ge σ* band tail varies with the number of Ge-Ge bonds around each Ge vertex.

**Figure 3:**
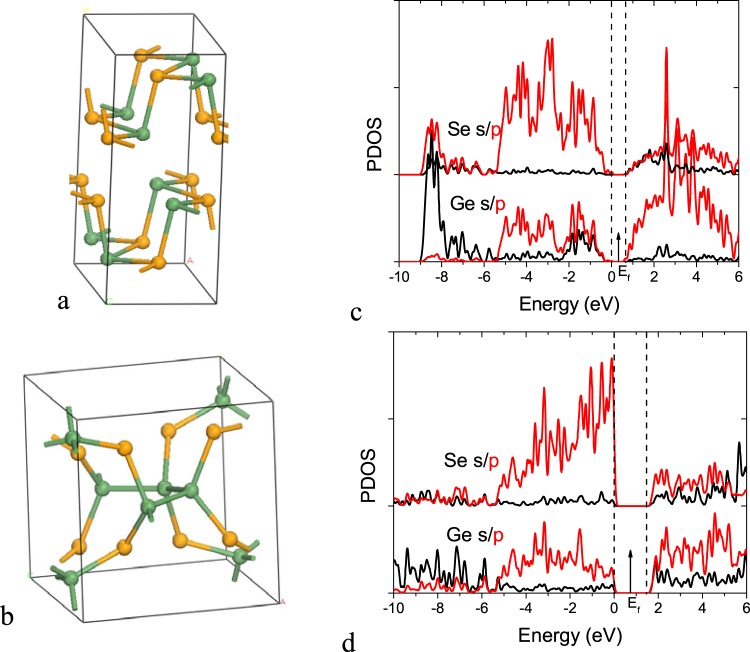
(**a**) The 3:3 bonded orthorhombic IV-VI structure, with only heteropolar bonds. (**b**) Ge-rich GeSe_2_ structure used for 4:2 bonded chalcogenide networks. (**c**) Partial density of states (PDOS) of orthorhombic structure for GeSe. (**d**) PDOS of the ordered 4:2 bonded GeSe network with Ge-Ge bonds in (**b**). Green balls = Ge, Orange balls = Se.

Our model is supported by detailed density functional calculations on supercell models. First, note that the band structure of the covalently-bonded chalcogenides differs considerably from that of the crystalline GeTe and GeSbTe compounds. For these, the band gap is narrow and arises from a Peierls distortion^9,10^ (Fig. 3a,b). On the other hand, bonding in a-GeSe_2−x_ is simple electron-pair covalent bonding, leading to wider gaps (Fig. 3c,d).

**Figure 4:**
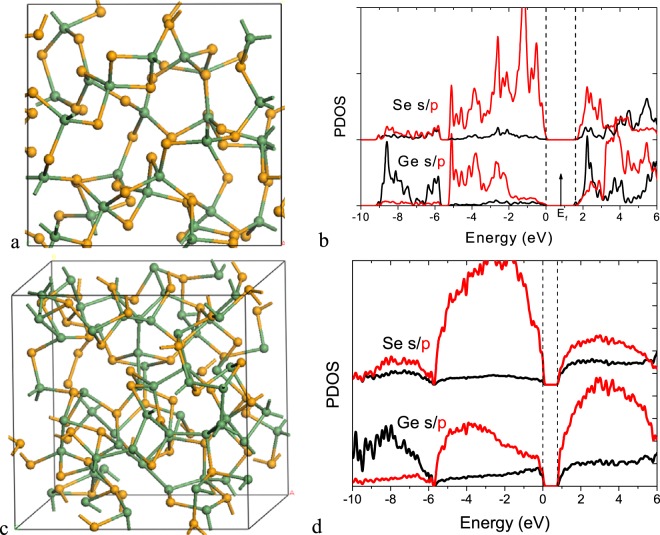
(**a**) Random network of amorphous GeSe_2_ with 4:2 bonded structure. (**b**) PDOS of the a-GeSe_2_ network in (**a**). (**c**) Random network of 4:2 bonded GeSe with numerous Ge-Ge bonds, and (**d**) PDOS of this structure. Green balls = Ge, Orange balls = Se.

We take a-GeSe_2_ with divalent Se sites as an example. Figure 4(a) shows a chemically ordered random network model of 4:2 bonded a-GeSe_2_ where each Ge site has four Ge-Se bonds. Figure 4(b) shows the partial density of states (PDOS) for this structure. It has a band gap of 1.7 eV in GGA. The calculated DOS here are consistent with the experimental spectra, measured by UPS, XPS and inverse photoemission for the case of the conduction band^21,32–35^. The upper valence band has three peaks at −1.4 eV, −3.0 eV and −4.6 eV as seen in UPS. Finally the peak at -9 eV corresponds to the Ge s states. The Se s states lie below the diagram. In the conduction band, the first peak is due to Ge s and Se p states, and the second is due to Ge p and Se p states.

Figure 4(c) shows a chemically disordered random network of 4:2 bonded a-GeSe with some Ge-Ge bonds. Figure 4(d) shows its PDOS. The lower CB peak consists mainly of Ge p-like states.

**Figure 5:**
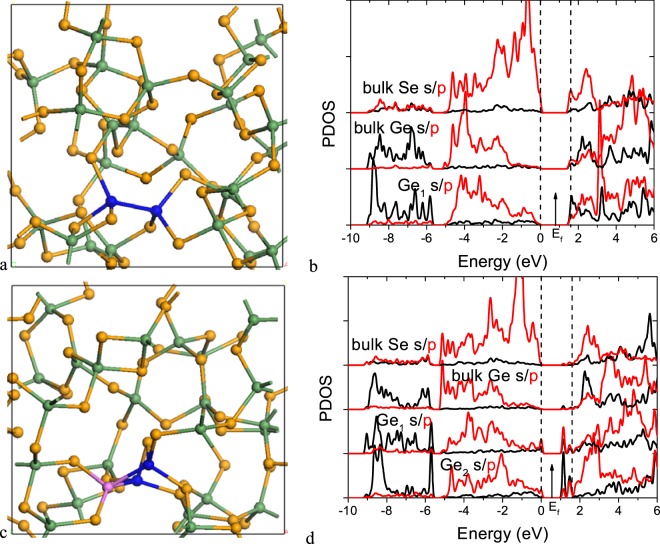
(**a**) GeSe_2−x_ network with single Ge-Ge bond. (**b**) PDOS of the network and the Ge site on the Ge-Ge bond in (**a**). (**c**) GeSe_2−x_ network with two Ge-Ge bonds meeting at a common Ge vertex. (**d**) PDOS of the network and two distinct Ge sites on the Ge-Ge bonds in (**c**), one at the end and another at junction of the two Ge-Ge bonds.

Figure 5(a) shows a chemically ordered 4:2 coordinated network of GeSe_2_ but with the addition of one Ge-Ge bond. This is formed by removing a Se site from a Ge-Se-Ge bridge and joining the two Ge sites together. While this process would make a gap state in a crystalline model, there are no gap states in the network due to relaxation, as the Ge-Ge σ* state lies just above the CB edge, Fig. 5(b).

Figure 5(c) shows a chemical ordered GeSe_2_ random network, but including two Ge-Ge bonds with a common vertex. Figure 5(d) shows its PDOS. This network now has gap states corresponding to Ge vertex sites. This behavior occurs generally. As noted earlier, the effective width of a Ge-Ge sub-band varies with the coordination number of the Ge-Ge sub-network. This causes the Ge-Ge bonds to form a distribution of tail states that extend below the conduction band edge. Clima^25^ finds that such states have a large Bohr radius and a large localization or inverse participation ratio (IPR) due to their Ge p-like character. Thus overall, our calculations show that a Ge excess within a overall 4:2 coordinated network causes a tailing of localized tail states below the conduction band edge, which will be available to create a non-linear conductivity at high applied fields.

**Figure 6:**
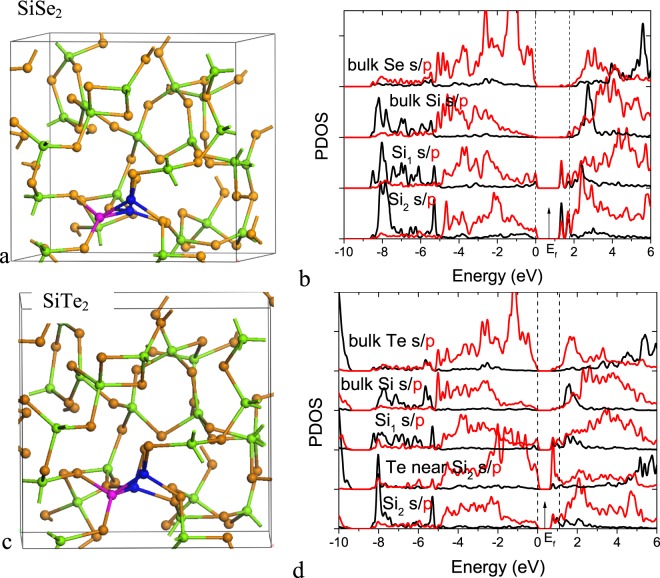
(**a**) SiSe_2−x_ network with two Si-Si bonds meeting at a common Si vertex. (**b**) PDOS of the network and two distinct Si sites on the Si-Si bonds, (**c**) SiTe_2−x_ network, with two Si-Si bonds meeting at common vertex. (**d**) PDOS of Si vertex.

Figure 6(a,b) show the network and PDOS for a SiSe_2−x_ network with Si-Si bonds, and Fig. 6(c,d) shows the network and PDOS of SiTe_2−x_ with Si-Si bonds. These have the same band layout as a-GeSe_2−x_, but with SiTe having a narrower gap and SiSe having a wider gap (Table 2). The same gap states appear. However, the longer bonds of SiTe cannot carry the high maximum current densities as GeSe, whereas the wider gap SiSe will mean that the non-linearity occurs at too large applied fields. Thus GeSe comes out as having the optimum properties.

**Table 2:** Calculated Band gaps of various stoichiometric 4:2 coordinated networks.

Compound	Band gap (eV)
GeSe_2_	1.60
SiSe_2_	1.78
SiTe_2_	1.10

We now consider how we can illustrate these trends in terms of phase stability maps. Phase stability maps were first used by Philips and VanVechten^36^ for the structural classification of the average valence-4, sp-bonded AB binary compounds. They used the covalent gap and ionic gap parameters derived from optical spectra to separate the covalent zincblende compounds from the ionic rocksalt compounds. The separation was improved by Chelikowsky and Philips^37^ who used the quantum defect orbital radii of StJohn and Simons^38^ to define two parameters rπ^−1^ (hybridization) and rσ (ionicity), which acted like the covalent gap and ionic gap, to separate out the zincblende, wurzite, rocksalt and CsCl structures.

The PCM and OTS materials are based on the 5-electron IV-VI binary compounds. Their bonding is primarily between p orbitals not sp orbitals as in 4-electron compounds. Thus, Littlewood^39^ defined slightly different parameters, with rπ^−1^ as before but the rσ parameter involving just p orbitals. These axes led to a structural separation of the rhombohedral, orthorhombic and rocksalt phases of the 5-electron binaries.

Lencer^40^ then laid the rπ^−1^, rσ coordinates of the high performing PCM compositions onto this phase map. It showed that PCMs were favored where the stable crystal phase has rhombohedral structure which supports resonant bonding^9,10^. We now extend this analysis method to treat selector materials and provide a more detailed understanding of the band structure design that leads to their non-linear conductivity behavior.

To treat the selector materials, we must extend the range of possible structures for the 5-electron semiconductors to higher rπ^−1^ values by including the covalent 4:2 coordinated SiO_x_ (silica-like) phase. Figure 4(a,b) shows the orthorhombic structure of GeSe containing only heteropolar bonds and Fig. 4(c,d) shows the silica-like phase of GeSe containing also homopolar Ge-Ge bonds^41^.

**Figure 7:**
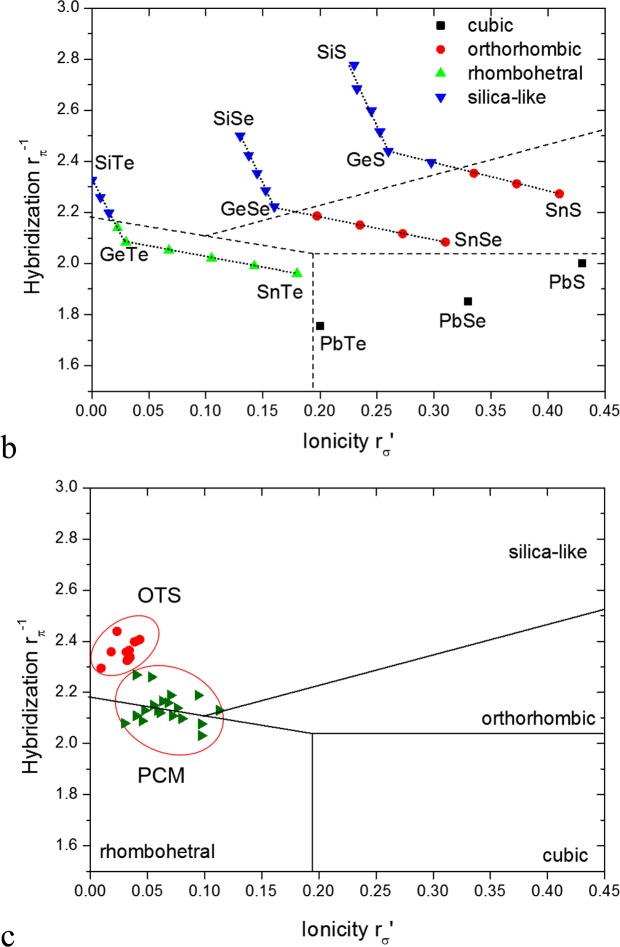
(**a**) Calculated phase stability diagram of the IV-VI compounds, including the 4:2 coordinated ‘silica-like’ phase of Fig. 3(b). Many (Ge_x_Si_1−x_)Te alloys were used to determine precise locations of phase boundaries. (**b**) Experimental data points from Lencer^40^ for PCMs and Velea^5^ for OTS amorphous alloys added to phase stability maps.

The phase boundaries are found by calculating the total energies of the different phases in Fig. 7(a,b) using density functional theory at closely spaced compositions across the phase map. Figure 7(a) shows the resulting stability regions of various IV-VI materials. We see that the plot reproduces the previous separation of rhombohedral, orthorhombic and rocksalt structures. A fourth region now appears for the 4:2 bonded silica-like structures, which covers most of the large hybridization parameter. Note that by including closely spaced alloy compositions, we can define accurately where each phase boundary lies. Interestingly, we find that the silica/orthorhombic phase boundary is sloped.

We now lay the experimental data points for OTS compositions of Velea^5^ and PCM materials from Lencer^40^ onto this map, Fig. 7(b). We see that the PCM region overlaps the rhombohedral and silica-like region, rather than being fully in the rhombohedral region. This indicates a slight offset in the prediction properties of the quantum-defect parameters.

We see that the experimental OTS selector compositions lie within the silica-like region of Fig. 7(b). This arises as follows. The maximum current density is a key criteria for selectors. This is limited by electromigration or the breaking of network bonds, just as it is for interconnects^42^. Thus, a large current-carrying capacity requires stronger shorter bonds, and this requires a large hybridization parameter. This means moving to lighter, shorter bond-length elements like Se, S and Si. The larger hybridization parameter moves it away from tellurides and resonant bonding, towards lighter chalcogenides with more covalent bonding. This means better glass formers and slower atomic transition rates. The compounds also remain in the amorphous state to higher working temperatures^6,25^. Given that selectors still require rapid transitions, then they should not use a structural phase transitions as in PCRAMs, but use the intrinsically rapid electronic-only transitions. Thus we arrive at OTS materials. However, we should limit it to well-screened chalcogenides of moderate band-gap to avoid too slow responses due to slow charge trapping as in SiO_2_.

A weakness of the a-GeSe-type alloys as selectors is that their off-current is too high. This can be counteracted by alloying with N or C^43–49^ as is well known previously. The addition of N or C both widens the band gap, reducing off-current, and also reduces atomic diffusion and nucleation rates, so raising the crystallization temperature. Thus, the overall selection becomes a-Ge-Se-type systems, not pure a-Ge-Se.

Our results are also consistent with experiment values in more general terms, where these are known. Amorphous Se is found to have a low p-type mobility from its photoconductivity^16^. Amorphous GeSe is different. Thermopower data shows that a-GeSe is n-type for x = 0.50 and for Ge-rich compositions^17^. This is consistent with the defects being mainly on the Ge sites and related to the conduction band. It is also consistent with the photoconductivity data of Govoneanu^4^ who found defect states at 0.4–0.5 eV below the CB edge. At low fields, the conductivity activation energy ΔE is found to be roughly half the band gap, showing that conductivity is by activation from E_F_ to the CB mobility edge. At high fields, ΔE is small, consistent with hopping within a narrow, dense range of states near the CB mobility edge. This contrasts with the phase change materials, where in crystalline GeTe and GeSbTe, the Ge vacancy states pin E_F_ close to the valence band edge, making it overall p-type^50^, whereas in the amorphous phase, various not fully identified defects pin E_F_ near midgap^51^.

## Conclusions

We have studied the bonding characteristics of Ge-rich Ge_x_Se_1−x_ and similar systems as typical chalcogenide OTS materials. It is shown that the network structure of 4:2 coordinated group IV chalcogenide alloys have a low electron effective mass, sizable donor Bohr radii, and a large tail of localized conduction states, leading to both non-linear conduction and a high current carrying capacity as desired for OTS devices. The favored compositions are found to cluster in the region of the hybridization/ ionicity plot where the 4:2 coordinated silica-like phase is most stable. Thus the OTS materials belong to the range of larger hybridization parameter than the PCM materials, explaining their greater structural rigidity which is required for OTS operation. It is found that at the same time as retaining the 4:2 bonded structure, certain Ge-Ge bonding configurations create conduction band tail states that lead to the non-linear conduction. This work promotes a better understanding of the bonding origin of threshold switching in chalcogenides and allows optimization of selector devices.

## Methods

To calculate the phase boundaries, we carried out first-principles density functional theory calculations using the plane wave CASTEP code^52^. The generalized gradient approximation (GGA) was used for the exchange-correlation function. Corrections to the GGA description of van der Waals bonding were included using the Tkatchenko and Scheffler scheme^53^. A unit cell of different chalcogenide bonding structures are geometrically optimized with 1000 eV energy cut-off and dense k-point meshes with separation as close as 0.03 Å^−1^.
